# An Integrated Review of Psychological Stress in Parkinson's Disease: Biological Mechanisms and Symptom and Health Outcomes

**DOI:** 10.1155/2016/9869712

**Published:** 2016-12-12

**Authors:** Kim Wieczorek Austin, Suzanne Weil Ameringer, Leslie Jameleh Cloud

**Affiliations:** ^1^Virginia Commonwealth University School of Nursing, 1100 East Leigh Street, Richmond, VA 23219, USA; ^2^Virginia Commonwealth University Parkinson's and Movement Disorders Center and VCU Health Neuroscience, Orthopaedic, and Wellness Center, 11958 West Broad Street, Richmond, VA 23233, USA

## Abstract

Parkinson's disease (PD) is characterized by complex symptoms and medication-induced motor complications that fluctuate in onset, severity, responsiveness to treatment, and disability. The unpredictable and debilitating nature of PD and the inability to halt or slow disease progression may result in psychological stress. Psychological stress may exacerbate biological mechanisms believed to contribute to neuronal loss in PD and lead to poorer symptom and health outcomes. The purpose of this integrated review is to summarize and appraise animal and human research studies focused on biological mechanisms, symptom, and health outcomes of psychological stress in PD. A search of the electronic databases PubMed/Medline and CINAHL from 1980 to the present using the key words* Parkinson's disease and stress, psychological stress, mental stress, and chronic stress* resulted in 11 articles that met inclusion criteria. The results revealed significant associations between psychological stress and increased motor symptom severity and loss of dopamine-producing neurons in animal models of PD and between psychological stress and increased symptom severity and poorer health outcomes in human subjects with PD. Further research is needed to fully elucidate the underlying biological mechanisms responsible for these relationships, for the ultimate purpose of designing targeted interventions that may modify the disease trajectory.

## 1. Introduction

Parkinson's disease (PD) is characterized by complex symptom patterns that fluctuate in onset, severity, responsiveness to treatment, and associated level of disability. The classic motor symptoms of tremor, rigidity, bradykinesia, and postural instability are compounded by nonmotor symptoms such as depression, cognitive impairments, sleep disturbances, fatigue, pain, and autonomic dysfunction. Many of these nonmotor symptoms respond poorly to available treatment options and significantly contribute to poorer quality of life and increased functional disability [1]. Early in the course of the disease, medications for PD typically improve motor symptoms. However, as the disease progresses, higher medication doses become necessary which can then cause debilitating dystonia and dyskinesia [2]. Reductions in medication dosages are often required to lessen the severity of these complications, resulting in breakthrough primary motor symptomology. Further complicating the illness experience, prolonged and/or high dose treatment with PD medications has been associated with on-off phenomena, which leads to unpredictable motor symptom exacerbations and periods of immobility. The ability to reduce these medication-induced complications while still achieving motor symptom benefits becomes more difficult as the disease progresses, leading to greater functional disability and poorer quality of life.

The unpredictable and debilitating nature of the symptoms associated with PD combined with the inability to halt or slow disease progression has the potential to result in psychological stress. Psychological stress is a complex phenomenon that involves cognitive, emotional, behavioral, and biological responses to events or experiences that are perceived as threatening [3]. An individual's ability to cope with and adapt to psychological stress can be influenced by the number and significance of stressful events experienced within a given period of time, the degree to which stressors are perceived as threatening or harmless, and biological responses designed to promote adaptation [4, 5]. The inability to cope with or adapt to psychological stress has been associated with poorer symptom and health outcomes that may be relevant in PD. For example, in non-PD populations, significant relationships have been demonstrated between psychological stress and increased motor symptomology [6], pain [7, 8], fatigue, [6, 8], cognitive decline [9], and functional disability [9, 10].

Biological responses of the neuroendocrine and immune systems represent plausible mechanisms that may explain relationships between psychological stress and poorer symptom and health outcomes. A complex network of bidirectional links between the neuroendocrine and immune systems serve as major regulatory mechanisms for mounting effective biological responses to psychological stress [11]. The hypothalamic-pituitary-adrenal axis (HPAA) coordinates these responses by stimulating the release of cortisol in humans and corticosterone in humans and rodents. Glucocorticoids play an important role in mediating immunological responses to psychological stress by regulating microglial activation and proinflammatory cytokine and transcription factor expression and release [12]. Stress-induced dysregulation of relationships between the neuroendocrine and immune systems has been associated with neuroinflammation, oxidative stress, and loss of dopamine (DA) producing neurons within the central nervous system [13–18]. Prolonged exposure to psychological stress has also been shown to sensitize the neuroendocrine and immune systems to the detrimental effects of future insults, thereby exaggerating inflammatory responses, oxidative stress, and neuronal loss [14, 19, 20].

Based upon work in non-PD animal models, a number of underlying biological mechanisms have been implicated in stress-induced neuroinflammation, oxidative stress, and neuronal loss to include microglial activation, upregulation of proinflammatory cytokines, transcription factors, and isoenzymes, increased production of reactive oxygen species (ROS), and imbalances in the production of ROS and antioxidant reduction.  Table 1 summarizes the literature on these biological mechanisms. Specifically, studies have demonstrated that both stress and the administration of exogenous corticosterone increase microglial activation, reactivity, and proliferation [14, 16, 20]. Under normal conditions, microglia cells, which are found in particularly high concentrations in the substantia nigra, exist in a resting state. Once activated, microglia provide the first line of immune defense by releasing immune mediators that coordinate innate and adaptive immune responses within the central nervous system to include the expression and release of proinflammatory cytokines and transcription factors [21, 22]. Whereas acute activation of microglia facilitates tissue repair, prolonged or exaggerated responses result in increased neuroinflammation and oxidative stress, both of which have been associated with dopaminergic neurotoxicity [14, 21–23]. Dopaminergic neurons are particularly vulnerable to neuroinflammatory and oxidative processes due to the high rate of oxygen consumption and limited antioxidant defenses within the central nervous system [24].

The upregulation of proinflammatory cytokines and transcription factors may further perpetuate stress-induced neuroinflammation and oxidative stress within the central nervous system. Cytokines play an important role in stimulating and coordinating the cellular interactions necessary for mounting effective immune responses to infection, injury, and disease [25]. Psychological stress has been associated with significant elevations in tumor necrosis factor alpha (TNF-*α*), interleukin-1 (IL-1), and interleukin-6 (IL-6), proinflammatory cytokines that have been implicated in oxidative stress and apoptosis within the central nervous system [26–29]. Proinflammatory cytokines also stimulate activation of nuclear transcription factor-kappa B (NF-kB) pathways [30, 31], which have been implicated in apoptosis of nigral dopaminergic neurons in animal models of PD [17, 32]. Activation of NF-kB pathways also results in the production of cyclooxygenase-2 (COX-2), an enzyme involved in prostaglandin mediated inflammatory responses [33]. While COX-2 is normally expressed in relatively constant amounts within the central nervous system, stress-induced upregulation of COX-2 has been associated with increased generation of ROS, oxidative stress, neurotoxicity, and apoptosis within the central nervous system [30, 34, 35].

Stress-induced oxidative mechanisms have also been associated with neuronal loss within the central nervous system. Oxidation, and the subsequent production of ROS, occurs normally throughout the body as a result of aerobic metabolism. The greatest concentrations of ROS are found within mitochondria, the main site of adenosine triphosphate (ATP) production [36]. Under normal conditions, ROS are maintained at relatively constant levels as a result of balances between the rate of ROS production and removal by antioxidant substrates [37]. Oxidative stress occurs as a result of imbalances in the production of ROS and antioxidant reduction, resulting in excessive lipid peroxidation and tissue injury within the central nervous system. Psychological stress has been associated with increased mitochondrial production of ROS [38] and increased production of nicotinamide adenine dinucleotide phosphate (NADPH) oxidase, a membrane-bound enzyme involved in neutrophil respiratory bursts [39]. Normally, NADPH remains latent in neutrophils and is tightly regulated by hormones, cytokines, and a variety of other mechanisms. Stress-induced activation of NADPH has been associated with increased production of ROS, neuroinflammation, and degeneration of dopaminergic and nondopaminergic neurons [39]. Psychological stress has also been associated with decreased expression and release of antioxidants [38, 40]. Dysregulation of the antioxidant system, coupled with increased ROS production, has been shown to perpetuate oxidative stress, lipid peroxidation, and tissue damage within the central nervous system [26, 38, 41].

Stress-induced dysregulation of the neuroendocrine and immune systems may play an important role in symptom and health outcomes in PD. While the exact cause remains unknown, progressive loss of dopaminergic neurons within the substantia nigra pars compacta (SNc) and nondopaminergic neurons within the central, peripheral, and autonomic nervous systems are believed to contribute to symptom and disease progression [42]. As briefly summarized in Table 2, a considerable body of research exists to suggest that biological mechanisms associated with neuroinflammation and oxidative stress contribute to the loss of dopaminergic neurons in PD. These biological mechanisms include but are not limited to increased microglial activation [43–45], atypical production of select proinflammatory cytokines (IL-1*β*, IL-6, TNF-*α*, and IFN-*γ*) [41, 46, 47], enhanced activation of NF-*κ*B pathways [32, 48], increased upregulation of COX-2 [49] and NDAPH oxidases [50], and increased production of select biomarkers of oxidative stress [51–53]. As such, stress-induced neuroendocrine and immune system dysregulation may exacerbate pathogenic mechanisms in PD, resulting in poorer symptom and health outcomes. The purpose of this integrated review is to summarize and critically appraise the current state of the science regarding biological mechanisms and symptom and health outcomes of psychological stress in individuals with and animal models of PD. Limitations of the existing literature as well as directions for future research will also be discussed.

## 2. Methods

**Figure 1:**
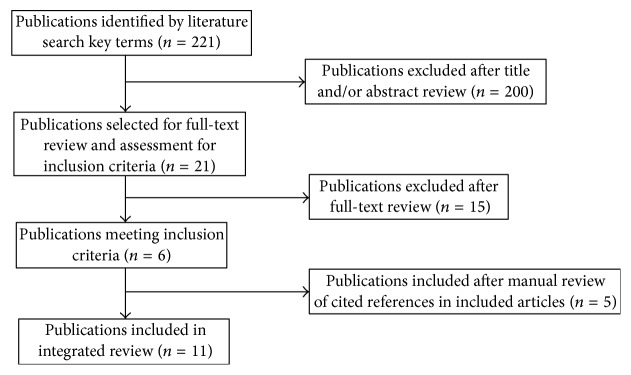
Literature review process.

An integrated review was conducted to examine human and animal research studies that focused on biological mechanisms and symptom and health outcomes of psychological stress in PD. A title search of the electronic databases PubMed/Medline and CINAHL was conducted from 1980 to the present using the key words* Parkinson's disease and stress, psychological stress, mental stress, and chronic stress*. A total of 221 articles were identified (Figure 1). Articles were reviewed based on the following inclusion criteria: (1) research studies involved human subjects with PD or animal models of PD with a primary aim of examining the effects of psychological stress on biological mechanisms and symptom and health outcomes; (2) animal studies reported using a recognized model of PD to include 1-methyl-4-phenyl-1,2,3,6-tetrahydropyridine (MPTP), 6-hydroxydopamine (6-OHDA), or rotenone induction; (3) animal studies involved the induction of psychological stress; (4) human studies involved the induction of psychological stress and/or quantified psychological stress levels; and (5) the study was written in English. Based on these inclusion criteria, a title/abstract review resulted in the exclusion of 200 articles, the majority of which focused on oxidative, nitrosative, and/or endoreticulum stress (*n* = 171) or caregiver stress (*n* = 9) in PD. Full-text review and assessment for inclusion criteria was conducted on the remaining 21 articles. An additional 15 articles were excluded, the majority of which were literature reviews and involved biological mechanisms of psychological stress in non-PD animal models or case-studies that did not involve the induction of psychological stress and/or quantify psychological stress levels. The remaining six articles met the above identified inclusion criteria and were included in this review. An additional five articles were identified after a manual review of the references cited in the included articles and excluded articles that underwent a full-text review. A total of eleven articles met the inclusion criteria and were included in this review.

## 3. Results and Discussion

Of the 11 studies included in this review, seven examined biological mechanisms of psychological stress that contribute to pathophysiological processes and symptom outcomes in animal models of PD [55–61]. In contrast, the remaining four studies focused on biological mechanisms, symptom, and/or health outcomes of psychological stress that may modify the illness trajectory in human subjects with PD [62–65]. Each of these studies is further discussed below.

### 3.1. Biological Mechanisms of Psychological Stress That Contribute to Pathophysiological Processes and Symptom Outcomes in Animal Models of PD

Seven of the studies included in this review examined biological mechanisms and symptom outcomes of psychological stress in animal models of PD [55–61]. Health outcomes of psychological stress were not examined in these studies. Biological mechanisms in the reviewed studies included biomarkers of dopamine production and metabolism [55, 56, 58–61], serotonin (5-HT) [55], norepinephrine [61], and/or dopaminergic neurodegeneration [57, 58]. In addition, two studies examined biological mechanisms associated with stress responses of the neuroendocrine system, specifically the effects of corticosterone administration [57] and norepinephrine levels [61] on motor symptom outcomes. Of particular importance, one study [55] examined the effects of psychological stress on the expression of *α*-synuclein, a misfolded protein complex that is recognized as a pathological hallmark of PD. All of the animal studies included in this review examined symptom outcomes specific to the motor manifestations of PD [55–61]. Only one study examined the effects of psychological stress on behaviors associated with depression, a common nonmotor symptom in PD [55]. In addition, one study focused on the effects of psychological stress on the neuroprotective effects of voluntary exercise on symptom and biological outcomes [58]. These studies are further discussed below and summarized in Table 3.

Using a MPTP/probenecid (MPTP/p) animal model of PD, researchers examined the effects of chronic mild psychological stress on biological mechanisms involving DA and 5-HT levels and the expression of dopaminergic markers of the nigrostriatal (substantia nigra and striatum) and nonnigrostriatal (hippocampus, cortex, and cerebellum) systems and symptom outcomes specific to depression [55]. Symptom outcomes of depression included measures of locomotion, activity, and anhedonia. Subjects were randomly assigned to a saline-treated control group, MPTP/p group, stress group, MPTP/p followed by stress group, stress followed by MPTP/p group, and stress before and after MPTP/p group. Both the before and after MPTP/p stress groups demonstrated greater DA depletion in all studied brain regions when compared to the stress or MPTP/p alone groups, with greater levels of DA depletion identified in the MPTP/p followed by stress group. Serotonin levels were also decreased in both the stress and MPTP/p groups, with the greatest reduction identified in all studied brain regions in the MPTP/p followed by stress group. In the MPTP/p followed by stress group, greater reductions in tyrosine hydroxylase (TH), dopamine transporter (DAT), and vesicular monoamine transporters (VMAT-2) expression were identified in all studied brain regions, suggesting that stress affects the biosynthesis and transport of DA. In stress-treated MPTP/p subjects, stress exaggerated the expression of nigrostriatal and nonnigrostriatal *α*-synuclein, which plays a major role in the development and progression of PD. While no significant changes were identified in locomotion, activity, and anhedonia in the stress or MPTP/p groups, greater behavioral changes in all parameters were identified in the MPTP/p followed by stress group. Cumulatively, these findings provide important evidence to suggest that psychological stress may contribute to biological mechanisms, symptom outcomes, and disease progression in PD.

In a 6-OHDA animal model of PD, researchers examined the effects of chronic variable psychological stress on biological mechanisms involving DA as measured by TH cell counts and motor symptom outcomes [56]. Subjects were assigned to 6-OHDA lesioned stressed and nonstressed groups, a sham-lesioned group, and a control group. Both 6-OHDA groups demonstrated impaired contralateral forelimb use when compared to the sham-lesioned group. However, following four weeks of chronic, variable stress, the 6-OHDA lesioned group demonstrated significantly more forelimb asymmetry when compared to the 6-OHDA lesioned nonstressed group. Significantly lower TH cell counts were identified in the SNc of stressed 6-OHDA subjects when compared to nonstressed 6-OHDA subjects. These findings suggest chronic variable stress may exacerbate motor symptom outcomes as a result of biological mechanisms associated with dopamine deficiencies in PD.

In a 6-OHDA animal model of PD, researchers examined the effects of restraint stress and corticosterone administration on biological mechanisms associated with dopaminergic neurodegeneration and motor symptom outcomes [57]. The results revealed psychological stress and elevated corticosterone levels in lesioned subjects impaired skilled limb reaching and limb coordination, impeded spontaneous recovery and compensation, and altered exploratory behavior. Fluoro-Jade positive cells, a biomarker of neuronal degeneration, were detected earlier in stress-treated lesioned subjects than controls. In stress- and corticosterone-treated lesioned subjects, the loss of TH positive cells, a biomarker of dopamine-producing cells, was associated with a significant increase in Fluoro-Jade positive cells in the SNc. Stress- and corticosterone-treated lesioned subjects demonstrated significant reductions in Nissl-positive cells in the ventral tegmental area (VTA) and SNc, suggesting greater neurodegeneration in these areas when compared to controls. Stress- and corticosterone-treated lesioned subjects also demonstrated enhanced glial fibrillary acidic protein (GFAP) immunoreactivity in the SNc when compared to controls, indicating greater reactive gliosis in the central nervous system. In stress-treated lesioned subjects, motor impairments were associated with higher numbers of Fluoro-Jade positive cells in the SNc and VTA. Cumulatively, these findings provide important evidence to suggest that psychological stress and stress-response hormones may contribute to pathogenic mechanisms involved in motor symptom outcomes in PD as a result of biological mechanisms associated with dopaminergic neurodegeneration.

Using a 6-OHDA animal model of PD, researchers examined the effects of psychological stress on the neuroprotective effects of voluntary exercise to include motor symptom outcomes as measured by rotational behavior and biological mechanisms associated with dopaminergic neurodegeneration [58]. Subjects were randomly assigned to one of three groups: runners or stressed runners, both of which had free access to running wheels, or nonrunners who had immobilized running wheels. Nonrunners demonstrated significantly higher numbers of apomorphine-induced contralateral rotations, a measure indicative of DA depletions exceeding 80%, when compared to stressed and nonstressed runners. This finding suggests voluntary exercise exerted neuroprotective effects on dopaminergic neurons in both stressed and nonstressed runners. The administration of apomorphine resulted in a significant increase in rotational behavior in stressed runners when compared to nonstressed runners, suggesting that stress may ameliorate the neuroprotective effects of voluntary exercise. Nonstressed runners demonstrated a nonsignificant decrease in the percentage of dopaminergic neurons lost when compared to both the stressed runners (4%) and nonrunners (14%). Significant differences were also identified in the number of baseline wheel rotations and the amount of DA destruction in the stressed runners and between the stressed and nonstressed runners. Significant group differences were found in the number of apomorphine-induced rotations and loss of DA between the stressed and nonstressed runners, the stressed runners and nonrunners, and all three groups. These findings suggest psychological stress may cancel the neuroprotective effects of voluntary exercise and contribute to greater DA deficiencies and poorer motor symptom outcomes.

In a 6-OHDA animal model of PD, researchers examined the extent to which motor symptom outcomes as measured by akinetic and cataleptic behaviors and biological mechanisms specific to DA concentrations were affected by psychological stress [59]. Biological mechanisms investigated in this study included the extent to which nigrostriatal DA neurons were capable of responding to the additional demand for DA during tail-shock stress and relationships between extracellular striatal DA concentrations and motor symptom outcomes before and after psychological stress exposures. When compared to baseline, subjects exposed to tail-shock stress demonstrated significantly increased striatal extracellular DA, dihydroxyphenylacetic acid (DOPAC), and homovanillic acid (HVA) levels. These findings suggest residual nigrostriatal DA neurons are capable of producing DA in response to psychological stress. However, in all but one subject, these levels did not reach levels comparable to those demonstrated in nonlesioned animals following tail-shock stress. While no consistent pattern was demonstrated between stress and akinetic and cataleptic motor behaviors, a significant negative correlation was shown between poststress latencies for catalepsy and extracellular DA concentrations, with a similar trend identified for akinesia. These findings suggest psychological stress may lead to poorer motor symptom outcomes as a consequence of lower than normal DA responsiveness in the striatum.

In a MPTP animal model of PD, researchers examined the effects of immersion immobilization stress on motor symptom outcomes associated with locomotor activity and biological mechanisms involving DA content, metabolites (DOPAC and HVA), and indices (DOPAC + HVA/DA) [60]. Subjects were allocated to one of four subgroups: MPTP-treated group, saline-treated group, MPTP and stress-treated group, or saline and stress-treated group. The results revealed the induction of psychological stress in the MPTP-treated group was associated with a more pronounced but transient decrease in locomotor activity when compared to the stress-treated saline group. Striatal DA content was significantly lower in the stress-treated MPTP group when compared to the MPTP-treated group. Striatal DA indices were significantly elevated in both the MPTP- and saline-treated stress groups, indicating increased DA turnover. There was no significant difference in the striatal DA metabolites DOPAC or HVA between the two MPTP-treated groups or the two saline-treated groups. These findings provide evidence to suggest that psychological stress may contribute to motor symptom outcomes and biological mechanisms associated with dopamine deficiencies in PD.

Finally, in a 6-OHDA animal model of PD, researchers examined the effects of psychological stress on biological mechanisms involving DA and norepinephrine that may contribute to motor symptom outcomes of akinesia [61]. The results revealed the induction of acute psychological stress precipitated the development of transient akinesia in all lesioned subjects but had no effect on controls. Dopamine deficiencies in the striatum were more predictive of stress-induced akinesias than in other areas of the brain. While some subjects demonstrated moderate depletion in hippocampal norepinephrine levels, there was no consistent relationship with stress-induced akinesia. These findings suggest DA deficiencies may affect the ability to maintain normal motor function under conditions of psychological stress.

Collectively, these studies suggest important relationships exist between psychological stress and biological mechanisms that contribute to pathophysiological processes and symptom outcomes in animal models of PD. Specifically, significant relationships have been demonstrated between psychological stress and increased motor symptom severity [55–61] and depression [55] as well as dopamine deficiencies [55, 56, 58–61], dopaminergic neurodegeneration [55, 57], and the expression of *α*-synuclein [55]. One study demonstrated significant relationships between corticosterone administration, a key mediator of neuroendocrine stress responses, and increased motor symptoms and loss of dopaminergic neurons [57]. However, another study failed to demonstrate a significant relationship between hippocampal norepinephrine levels, another biomarker of neuroendocrine-mediated stress responses, and stress-induced akinesia [61], suggesting additional research is needed in order to fully elucidate the role the neuroendocrine system plays in biological mechanisms and symptom outcomes of psychological stress in PD. None of the reviewed studies examined underlying biological mechanisms of neuroinflammation and oxidative stress that may contribute to symptom and health outcomes in PD.

### 3.2. Symptom and Health Outcomes of Psychological Stress That May Modify the Illness Trajectory in Human Subjects with PD

The remaining four studies in this review examined symptom and health outcomes of psychological stress that may modify the illness trajectory in human subjects with PD [62–65]. Three of these studies focused on symptom outcomes of psychological stress to include freezing of gait (FoG) [63], the ability to experience pleasure, reach-to-grasp movements [64], and nonmotor symptom frequencies [65]. One study examined relationships between psychological stress and health outcomes [65]. In contrast to research in animal models of PD, only one study examined biological outcomes of psychological stress, specifically sympathetic skin responses (SSR), a biomarker of sympathetic cholinergic sudomotor function associated with autonomic dysfunction [62]. Each of these studies is further discussed below and summarized in Table 4.

In subjects with PD (*n* = 29) and controls (*n* = 27), researchers examined the effects of psychological stress on biological mechanisms of autonomic dysfunction, specifically SSR [62]. SSR is a noninvasive biomarker of sympathetic cholinergic sudomotor function associated with autonomic dysfunction. Autonomic dysfunction is common in PD and can result in nonmotor symptoms such as diaphoresis, hypotension, and urinary and gastrointestinal dysfunction. In this study, SSR onset latencies, peak-to-peak, and amplitude recordings were obtained before and after a series of mental stressors designed to induce psychological stress. The results revealed no significant difference in SSR parameters before or after mental stress between subjects with PD and controls. However, the exclusion of subjects presenting with clinical autonomic dysfunction may explain the lack of significant findings in this study.

In a cross-sectional study, researchers examined factors that influence symptom outcomes of FoG in subjects with PD (*n* = 130) [63]. FoG, a common motor symptom of PD, results in a sudden and transient inability to walk typically in response to obstacles or situations that affect visual or proprioceptive input. Early in the disease, FoG is often triggered when initiating walking or by turning, confined spaces, rushed situations, or approaching a destination. As the disease progresses, FoG begins to occur in the absence of these triggers, perpetuating the unpredictability of this symptom. While turning around and fatigue were the most prevalent factors cited as contributing to FoG, 53.1% of subjects (*n* = 105) cited being in a stressful situation as a significant trigger of FoG.

In subjects with PD (*n* = 19) and matched controls (*n* = 19), researchers examined the effects of psychological stress on symptom outcomes, specifically goal directed movements and hedonic responsiveness [64]. Impaired goal directed movements and reduced hedonic responsiveness, defined in this study as impaired reach-to-grasp movements and decreased ability to experience physical or social pleasure, respectively, are two common symptoms in PD. Subjective ratings of mood state and pleasure associated with eating as well as reach-to-grasp movement measurements were obtained at baseline and after the induction of emotional stress. Deterioration in mood and reduction in hedonic responsiveness following the induction of psychological stress were significantly more pronounced in subjects with PD than controls. Psychological stress did not result in significant differences in reach-to-grasp movements between subjects and controls. However, the majority of subjects in this study were receiving treatment with a combination of dopamine replacement therapy and dopamine agonists, which may have ameliorated the effects of psychological stress on motor symptoms.

In a cross-sectional study, researchers examined patterns of psychological problems in subjects with PD (*n* = 3075) to include symptom and health outcomes of psychological stress [65]. Cluster analysis revealed four patterns of psychological problems: general low stress, general high stress, sexual and social problems, and nonsocial problems. Approximately two-thirds of the total subjects (69% of women; 67% of men) reported symptom increases with even small amounts of stress. Subjects with high stress reported greater frequencies of depressive moods, sleep disturbances, anxiety, sexual problems, and communications difficulties. Subjects with high stress also reported a greater frequency of symptom increases with even small amounts of stress as well as poorer health outcomes to include greater difficulty coping with PD, poorer social relationships, less enjoyment of life, and needing more psychological support.

These studies provide preliminary evidence to support relationships between psychological stress and poorer symptom and health outcomes in individuals with PD. Significant relationships were demonstrated between psychological stress and symptom outcomes to include increased FoG [63], decreased ability to experience pleasure [64], and increased symptom frequencies for select nonmotor symptoms such as depressive mood, sleep disturbances, anxiety, sexual problems, and communication difficulties [65]. Psychological stress was not associated with significant differences in reach-to-grasp movements between subjects and controls [64]. Greater psychological stress was also associated with poorer health outcomes such as difficulty coping with PD, poorer social relationships, less enjoyment of life, and the need for more psychological support [65]. Evidence is lacking regarding biological mechanisms of psychological stress that may contribute to symptom and health outcomes in human subjects with PD.

## 4. Conclusion

This integrated review supports the notion that psychological stress affects biological mechanisms and symptom and health outcomes in PD. Evidence in animal models has demonstrated that significant relationships exist between psychological stress and poorer symptom outcomes to include increased motor symptom severity and behaviors associated with depression. Significant relationships have also been demonstrated between psychological stress and biological mechanisms involving biomarkers of dopamine production and metabolism, serotonin, and dopaminergic neurodegeneration. Specifically, the evidence suggests psychological stress in animal models of PD results in greater DA depletions in the nigrostriatal and nonnigrostriatal systems, exaggerated expression of nigrostriatal and nonnigrostriatal *α*-synuclein, increased neurodegeneration of dopaminergic neurons as indicated by increased Fluoro-Jade positive cells and GFAP in the SNc, and decreased 5-HT levels. These findings suggest the induction of psychological stress in animal models of PD contributes to poorer motor symptom outcomes by exacerbating underlying pathogenic features associated with PD.

Preliminary evidence also exists to suggest psychological stress may play a role in symptom and health outcomes in individuals with PD. In human subjects with PD, evidence supports relationships between psychological stress and increased symptom severity and poorer health outcomes. Significant relationships have been demonstrated between psychological stress and poorer symptom outcomes to include increased FoG, depressive moods, sleep disturbances, anxiety, sexual problems, and communication difficulties and decreased hedonic responsiveness. Greater levels of psychological stress have also been associated with poorer health outcomes such as difficulty coping with PD, poorer social relationships, less enjoyment of life, and the need for more social support. Evidence is lacking regarding underlying biological mechanisms of psychological stress that contribute to these findings in human subjects with PD.

Cumulatively, the studies included in this review provide evidence of the potential importance of psychological stress to biological mechanisms and symptom and health outcomes in PD. It should be noted however that many of the reviewed studies demonstrated limitations that may affect the validity and generalizability of these findings. These limitations include issues associated with translating experimental outcomes in animal models to human populations, key differences in the primary outcomes examined, methods for inducing psychological stress, and underlying stress paradigms and study design and methodologies issues.

A number of limitations exist when attempting to translate outcomes of animal experimentation to human populations. Routine laboratory procedures and conditions, such as artificial and restricted housing environments, noise, human handling, and contagious anxiety, have been associated with elevations in stress-related biomarkers, factors that may confound experimental results [66, 67]. The complexity of human diseases is often difficult to replicate, resulting in discrepancies between animal models of select diseases and actual human conditions [66–68]. Animal models involve the induction of diseases in healthy, homogenous subjects that lack the many predisposing factors and comorbidities that contribute to disease development and progression in human populations [67, 68]. Differences in physiology, behavior, pharmacokinetics, and genetics may limit the ability to generalize experimental data from animal models to human populations [66, 67]. Experimental animal studies often lack fundamental aspects of study design that are required in human clinical trials, specifically randomization, blinding of research personnel, and sample size calculations, which may result in overestimated outcome effects [67, 68]. Furthermore, the small sample sizes typically used in animal experimentation often lead to underpowered studies, which may increase the risk of erroneously detecting treatment effects [67, 68]. Each of these issues confounds the ability to translate findings in experimental models to human populations with PD.

Key differences were identified in the primary outcomes examined in the reviewed studies. The majority of the animal studies included in this review focused on the effects of psychological stress on biological mechanisms that may contribute to pathophysiological processes in PD whereas the studies involving human subjects focused on the effects of psychological stress on symptom and health outcomes that may modify the illness trajectory. Extreme caution should be exercised when attempting to extrapolate biological outcomes in animal models as a means of explaining factors that modify symptom and health outcomes in those living with PD. As such, this highlights the need for additional research that focuses on biological mechanisms of psychological stress specific to underlying pathophysiological processes that may modify symptom and health outcomes in human populations with PD.

Important differences were demonstrated in the manner in which psychological stress was induced in animal models of and human subjects with PD. In the reviewed studies, the induction of psychological stress in individuals with PD involved performing strenuous mental tasks [62, 64]. In contrast, the induction of psychological stress in animal models of PD involved physiological insults such as glucodeprivation, osmotic diuresis, hypothermia, and painful stimuli [59–61]. Stress induction techniques used in animal models may result in the activation of biological pathways unrelated to psychological stress responses. For example, the dopaminergic system has been implicated in biological responses associated with pain perception and processing variability as well as central control of thermoregulation [69, 70]. Activation of these pathways may have confounded the ability to interpret outcomes of psychological stress in animal models of PD. Furthermore, the induction of stress in animal models of PD does not adequately reflect the multidimensional and dynamic nature of stress experiences in individuals living with PD. Each of these factors necessitates the use of caution when attempting to translate these findings to human populations with PD.

Key differences in acute versus chronic stress paradigms are important to consider when evaluating the studies included in this review. The underlying stress paradigms in the majority of the reviewed studies focused on the implementation of acute psychological stressors that varied in timing and type so as to limit predictability [55, 57–62, 64]. Only one study specifically focused on chronic variable stress [56]. Important differences exist in biological mechanisms responsible for mediating physiological responses to acute versus chronic psychological stressors. For example, activation of the HPAA and the release of glucocorticoids in response to acute psychological stress have been associated with transient immunosuppressive effects [22, 71, 72]. In contrast, sustained activation of the HPAA and release of glucocorticoids, as seen with chronic psychological stress, have been associated with exaggerated inflammatory responses, cell damage, and death [18, 22, 73]. Chronic psychological stress has also been associated with dysregulation of glucocorticoid feedback mechanisms. Under normal circumstances, transient increases in circulating glucocorticoids exert inhibitory effects on further HPAA stimulation. Dysregulation of these feedback mechanisms, as seen with chronic psychological stress, has been associated with increased expression and release of glucocorticoids [18]. Furthermore, the increased energy demands needed to respond to chronic psychological stress have been associated with increased production of ROS, oxidative stress, lipid peroxidation, and tissue damage within the central nervous system [14, 26, 38, 74]. Given that individuals with PD often face a variety of stressors across the illness trajectory, additional research is needed that considers differences in biological mechanisms, symptoms, and health outcomes associated with acute versus chronic stress paradigms.

A number of design and methodological issues were identified in the reviewed studies. For instance, between-subject procedural variability [61], the introduction of potential confounders [58, 61], and inadequately operationalized key variables/procedures [56, 59, 60] were identified in several of the reviewed animal studies. With regard to the human studies, the limited number of studies and relatively small sample sizes in all but one study represent limitations to the generalizability of these findings. In several studies, inadequate operationalization of key variables and/or unknown psychometric properties for the measurement tools that were utilized may have affected the validity and reliability of the results [62–65]. The operationalization of hedonistic responsiveness as pleasurable food experiences in one study may also present a potential limitation given individuals with PD often experience loss of taste and swallowing difficulties, factors that were not controlled for and may have confounded differences in hedonistic responsiveness between subjects and controls [64]. Finally, in another study, advertising the study as a mobility study may have led to the overrepresentation of subjects with mobility problems [63].

As this review has demonstrated, significant gaps exist in our understanding of biological mechanisms and symptom and health outcomes of psychological stress in individuals living with PD. Much of what is currently known about biological mechanisms and symptom outcomes of psychological stress in PD has been conducted in animal models of PD and/or predicated on research in non-PD animal models. While important, this knowledge may not adequately reflect these constructs in individuals living with the disease, particularly given the multifaceted nature of stress in human populations and similarities in key pathogenic features of PD that may be exacerbated by underlying biological mechanisms of psychological stress. As a result, additional research is needed in order to further elucidate underlying biological mechanisms and symptom and health outcomes of psychological stress in PD. Psychoneuroimmunological frameworks provide an important opportunity for gaining insight into plausible biological mechanisms of the neuroendocrine and immune systems that may contribute to stress-induced neuroinflammation, oxidative stress, and loss of dopaminergic neurons in PD, which may lead to poorer symptom and health outcomes [11]. Rigorously designed studies that provide a deeper understanding of these relationships would provide the foundation for designing interventions specifically targeted to biological mechanisms and symptom and health outcomes of psychological stress in PD.

Healthcare providers need to be aware of multifaceted factors that may contribute to symptom and health outcomes in PD. Given the progressive and unpredictable nature of PD, individuals living with the disease must cope with and adapt to a variety of stressors over a protracted period of time. The cumulative costs of psychological stressors may further exacerbate pathogenic mechanisms in PD thereby perpetuating neuronal loss within the central nervous system. Consideration of these factors in the design of future research studies as well as the clinical care of individuals with PD represents an important opportunity to improve symptom and health outcomes in those living with the disease.

## Figures and Tables

**Table 1:** Overview of potential biological mechanisms of psychological stress-induced neuroinflammation, oxidative stress, and neuronal loss in non-PD animal models.

Study author/date	Major study findings
Lucca et al., 2009 [38]	Chronic mild psychological stress resulted in significant elevations in superoxide, a reactive oxygen species, in the submitochondrial particles of the prefrontal cortex, cortex, and hippocampus in subjects when compared to controls. The results also demonstrated significant elevations in TBARS, a measure of lipid peroxidation, in the cortex of stressed subjects.

De Pablos et al., 2006 [14]	Induction of chronic variate psychological stress enhanced LPS-induced neuroinflammation in the PFC of stressed subjects when compared to nonstressed LPS-induced subjects and controls. Significant findings included increased microglial activation, levels of DA and its metabolite DOPAC, expression of proinflammatory cytokine mRNA (TNF-*α*, IL-1*β*, and IL-6), activation of MAP kinases, and loss of NeuN-positive neurons in the PFC.

Munhoz et al., 2006 [18]	Chronic, unpredictable psychological stress potentiated NF-*κ*B binding activity in the frontal cortex and hippocampus and proinflammatory gene expression of IL-*β*, TNF-*α*, and NOS-2 as mediated by elevated GC levels in LPS-induced subjects when compared to controls.

Kim et al., 2005 [41]	Acute psychological stress resulted in elevated BH4 and DA levels in striatal tissues and led to greater lipid peroxidation, protein-bound quinone, neuromelanin, and antioxidant enzyme activities, markers of oxidative stress, in the substantia nigra and striatum of subjects when compared to controls. Furthermore, in subjects exposed to stress, TH-immunoreactive DA neurons demonstrated strong Fluoro-Jade staining, indicating selective degeneration of dopaminergic neurons. In contrast, no Fluoro-Jade staining was identified in controls.

Munhoz et al., 2004 [26]	Repeated psychological stress was associated with time-dependent markers of oxidative stress in brain tissue to include increase in Ca^2+^-independent NOS-2 activity, lipid peroxidation, TNF-*α*, and TACE activity in subjects when compared to controls.

Madrigal et al., 2003 [33]	Acute psychological stress was associated with higher levels of PGE_2_, a marker of COX-2 neuronal activity, MDA and oxidized glutathione, markers of lipid peroxidation, and NOS-2 in the cortex of subjects when compared to controls.

Madrigal et al., 2002 [30]	Acute psychological stress induced the expression of iNOS in the brain cortex, which was preceded by increased expression of TACE and the subsequent release of TNF-*α* in subjects as compared to controls. Furthermore, the results demonstrated that increased production of TNF-*α* was involved in stress-induced expression of iNOS as mediated by activation of NF-*κ*B.

TBARS, thiobarbituric acid reactive species; LPS, lipopolysaccharide; PFC, prefrontal cortex; DA, dopamine; DOPAC, 3,4-dihydroxyphenylacetic; mRNA, messenger ribonucleic acid; TNF-*α*, tumor necrosis factor alpha; IL-1*β*, interleukin-1 beta; IL-6, interleukin-6; MAP, mitogen-activated protein; NeuN-positive, neuronal nuclei positive; GC, glucocorticoids; BH4, tetrahydrobiopterin; TH-immunoreactive, tyrosine hydroxylase; Ca^2+^, calcium^2+^; NOS-2, inducible nitric oxide synthase; TACE, TNF-*α* converting enzyme; iNOS, inducible nitric oxide synthase; PGE_2_, prostaglandin E2; COX-2, cyclooxygenase-2; MDA, malondialdehyde.

**Table 2:** Potential biological mechanisms of neuroinflammation and oxidative stress associated with neurodegeneration in PD.

Study author/date	Major study findings
*Microglia activation*	
Gerhard et al., 2006 [43]	In vivo PET imaging revealed widespread and longitudinal microglial activation in subjects with PD when compared to controls
Ouchi et al., 2005 [44]	Microglial activation was associated with damage in nigrostriatal pathway in drug-naïve subjects with PD when compared to controls
Depino et al., 2003 [45]	Induction of PD in animals (6-OHDA model) resulted in increased microglial activation and atypical production of proinflammatory cytokine mRNA when compared to controls
*Proinflammatory cytokine production*	
Lindqvist et al., 2012 [54]	Serum levels of IL-6 significantly higher in subjects with PD than controls
Scalzo et al., 2010 [46]	Serum levels of IL-6 significantly higher in subjects with PD than controls
Reale et al., 2009 [47]	Basal and bacterial LPS-induced production of IL-1*β*, TNF-*α*, and IFN-*ϒ* significantly higher in subjects with PD than controls
*Proinflammatory transcription pathway activation*	
Tobón-Velasco et al., 2013 [48]	Induction of PD in animals (6-OHDA model) resulted in enhanced NF-*κ*B activation which was associated with increased TNF-*α* and COX-2 levels when compared to controls
Liang et al., 2007 [32]	Induction of PD in animals (6-OHDA model) resulted in activation of NF-*κ*B pathways which contributed to oxidative stress-induced degeneration of dopaminergic neurons when compared to controls
*Proinflammatory isoenzyme production*	
Hernandes et al., 2013 [50]	Induction of PD in animals (6-OHDA) demonstrated that NDAPH oxidases contribute to dopaminergic neurodegeneration in the nigrostriatal pathway
Teismann et al., 2003 [49]	Brain tissue samples of subjects with and animal models of PD (6-OHDA) demonstrated increased COX-2 upregulation in dopaminergic neurons when compared to controls
*Oxidative stress*	
Lin et al., 2012 [51]	Induction of PD in animals (rotenone model) associated with significantly higher levels of oxidative proteins in the striatum leading to greater levels of apoptotic cell death of dopaminergic neurons within the nigrostriatal system when compared to controls
Seet et al., 2010 [52]	Biomarkers of oxidative stress (F_2_-isoprostanes, hydroxyeicosatetraenoic acid products, 7B- and 27-hydroxycholesterol, 7-ketocholesterol, neuroprostanes, and urinary 8-hydroxy-2′deoxyguanosine) significantly higher in subjects with PD when compared to controls
Keeney et al., 2006 [53]	Misassembled mitochondrial complex I as reflected by significant loss of its 8 kDa subunits associated with oxidative damage in brain tissue of subjects with PD when compared to controls

PET, position emission tomography; PD, Parkinson's disease; 6-OHDA, 6-hydroxydopamine; mRNA, messenger ribonucleic acid; IL-6, interleukin-6; LPS, lipopolysaccharide; IL-1*β*, interleukin-1 beta; TNF-*α*, tumor necrosis factor alpha; IFN-*ϒ*, interferon gamma; NF-*κ*B, nuclear factor-kappa-light-chain-enhancer of activated B cells; COX-2, cyclooxygenase-2; NDAPH oxidase, nicotinamide adenine dinucleotide phosphate oxidase.

**Table 3:** Biological mechanisms of psychological stress that contribute to pathophysiological processes and symptom outcomes in animal models of PD.

Study author & date	Study purpose & sample	Method(s) of psychological stress induction	Measures of biological mechanisms & symptom outcomes	Major study findings
Janakiraman et al., 2016 [55]	*Purpose: *to examine the effects of psychological stress on symptom outcomes of depression and biological mechanisms of DA, 5-HT, and *α*-synuclein *Sample:* male C57BL/6 mice (*n* = 72); MPTP/probenecid induction	“Cage tilting, damp sawdust, placement in empty cage, group housing, placement in empty cage with water on the bottom, placement of a foreign object in cage, inversion of light/dark cycle, food or water deprivation, lights on for a short period of time during the dark phase, and switching cages” (p. 3)	*Biological mechanisms:* DA, 5-HT, TH, DAT, VMAT-2, *α*-synuclein levels in nigrostriatal (substantia nigra and striatum) and nonnigrostriatal tissues (hippocampus, cortex, and cerebellum) *Symptom outcomes:* depression as measured by behavioral deficits and anhedonia using the (a) open filed test; (b) narrow beam walking test; and (c) sucrose intake test	*Biological mechanisms: *increased depletion of DA, 5-HT, TH, DAT, and VMAT-2 was identified in stress-treated lesioned subjects. Stress exaggerated the expression of nigrostriatal and nonnigrostriatal *α*-synuclein. *Symptom outcomes: *stress increased behavioral deficits and anhedonia in stress-treated lesioned subjects.

Hemmerle et al., 2014 [56]	*Purpose: *to examine the effects of psychological stress on motor symptom outcomes and biological mechanisms associated with loss of DA neurons *Sample: *rats (sample size/gender not provided); 6-OHDA induction	Chronic variable stress (protocol not provided)	*Biological mechanisms: *TH cell counts in the SNc *Symptom outcomes: *forelimb asymmetry tests (not defined)	*Biological mechanisms: *stress was associated with significantly lower TH cell counts in the SNc in lesioned subjects. *Symptom outcomes: *stress was associated with significant forelimb asymmetry in lesioned subjects.

Smith et al., 2008 [57]	*Purpose: *to examine the effects of psychological stress and corticosterone administration on motor symptom outcomes and biological mechanisms of DA neurodegeneration *Sample:* female rats (*n* = 71); 6-OHDA induction	Restraint in Plexiglas tubes	*Biological mechanisms: *plasma concentrations of corticosterone and TH positive cells, Fluoro-Jade B cells, and GFAP immunoreactivity in the MTA, VTA, and SNc *Symptom outcomes: *skilled forelimb reaching, skilled walking, open field behavior, and apomorphine-induced rotations	*Biological mechanisms: *in stress- and corticosterone-treated lesioned subjects, the loss of TH positive cells was associated with significant increases in Fluor-Jade B cells in the SNc. Significant reductions in Nissl-positive cells in the VTA and SNc and enhanced GFAP immunoreactivity in the SNc were also demonstrated in stress-and corticosterone-treated lesioned subjects. *Symptom outcomes: *stress and elevated corticosterone levels impaired skilled limb reaching and limb coordination, impeded spontaneous recovery, and altered exploratory behavior in lesioned subjects.

Howells et al., 2005 [58]	*Purpose: *to examine the effects of psychological stress on the neuroprotective effects of voluntary exercise on motor symptom outcomes and biological mechanisms of dopaminergic neurodegeneration *Sample: *male rats (*n* = 31); 6-OHDA induction	Running wheel immobilization and shifting light/dark cycles	*Biological mechanisms: *TH positive cells in the SNc *Symptom outcomes: *number of running wheel revolutions and apomorphine-induced rotations	*Biological mechanisms: *stressed runners demonstrated lower TH positive cells in the SNc. *Symptom outcomes: *stressed runners demonstrated a significant increase in rotational behavior.

Keefe et al., 1990 [59]	*Purpose: *to examine the extent to which psychological stress affects motor symptom outcomes and biological mechanisms specific to DA concentrations *Sample:* male rats (sample size not provided); 6-OHDA	Tail-shock stress	*Biological mechanisms: *extracellular striatal DA, DOPAC, and HVA levels in vivo and in brain tissue specimens *Symptom outcomes: *akinesia defined as latency to move all four paws when placed on a flat surface within 120 seconds and catalepsy defined as latency to return all four paws to table surface within 120 seconds	*Biological mechanisms: *subjects exposed to tail-shock stress demonstrated significantly increased striatal extracellular DA, DOPAC, and HVA levels. In all but one subject, these levels did not reach levels comparable to those demonstrated in nonlesioned animals following tail-shock stress. *Symptom outcomes: *no consistent pattern was demonstrated between stress and akinetic and cataleptic motor behaviors. A significant negative correlation was shown between poststress latencies for catalepsy and extracellular DA concentrations, with a similar trend identified for akinesia.

Urakami et al., 1988 [60]	*Purpose: *to examine the effects of psychological stress on motor symptom outcomes and biological mechanisms involving DA *Sample:* male rats (*n* = 20); MPTP induction	Immobilization in water kept at 25°C for 15 consecutive hours	*Biological mechanisms: *striatal DA content, DOPAC and HVA levels, and DA indices (DOPAC + HVA/DA) in brain tissue specimens *Symptom outcomes: *locomotor activity (not defined)	*Biological mechanisms: *striatal DA content was significantly lower in stress-treated lesioned subjects. Striatal DA indices were significantly elevated in both the stress-treated lesioned and control groups. No significant difference was demonstrated in the DA metabolites DOPAC or HVA. *Symptom outcomes: *in lesioned subjects, stress was associated with more pronounced but transient decreases in locomotor activity.

Snyder et al., 1985 [61]	*Purpose: *to examine the effects of psychological stress on motor symptom outcomes of akinesia and biological mechanisms involving DA and norepinephrine levels *Sample: *male rats (*n* = 194); 6-OHDA induction	Glucodeprivation while withholding food, osmotic diuresis while withholding water, cold exposure, and tail shock	*Biological mechanisms: *DA and norepinephrine content in brain tissue specimens *Symptom outcomes: *akinesia defined as latency to move of greater than 60 seconds during the following two tests: (a) movement of all four paws when placed on a flat surface and (b) return of front or rear paws to the ground after being elevated on a Styrofoam block	*Biological mechanisms: *stress was associated with DA deficiencies in the striatum. *Symptom outcomes: *striatal DA deficiencies were more predictive of stress-induced akinesias than in other areas of the brain. There was no consistent relationship between hippocampal norepinephrine levels and stress-induced akinesia.

MPTP, 1-methyl-4-phenyl-1,2,3,6-tetrahydropyridine; DA, dopamine; 5-HT, serotonin; TH, tyrosine hydroxylase; DAT, dopamine transporter; VMAT-2, vesicular monoamine transporters; 6-OHDA, 6-hydroxydopamine; SNc, substantia nigra pars compacta; MTA, medial tegmental area; VTA, ventral tegmental area; GFAP, glial fibrillary acidic protein; DOPAC, dihydroxyphenylacetic acid; HVA, homovanillic acid.

**Table 4:** Symptom and health outcomes of psychological stress that may modify the illness trajectory in human subjects with PD.

Study author & date	Study purpose & sample	Method(s) of psychological stress induction	Measures of biological mechanisms, symptom, and health outcomes	Major study findings
Giza et al., 2012 [62]	*Purpose:* to examine the effects of psychological stress on biological mechanisms of autonomic dysfunction, specifically SSR parameters *Sample: *male and female subjects with PD and controls (*n* = 56)	Arithmetic calculations using the WAIS-R arithmetic subscale	*Biological mechanisms: *4-channel Nihon Kohen Neuropack S/MFrB 5504K used to record SSR in accordance with International Federation of Clinical Neurophysiology Guidelines	*Biological mechanisms: *no significant differences in SSR parameters were demonstrated between subjects or controls before or after the induction of psychological stress.

Rahman et al., 2008 [63]	*Purpose: *to examine factors that influence symptom outcomes of FoG in subjects with PD *Sample: *male and female subjects with PD (*n* = 130)	Not applicable	*Symptom outcomes: *factors influencing walking/freezing questionnaire (tool not specified)	*Symptom outcomes: *stress was identified as a trigger of FoG by 53.1% of subjects.

Macht et al., 2007 [64]	*Purpose: *to examine the effects of psychological stress on symptom outcomes, specifically goal directed movements and hedonic responsiveness *Sample: *male and female subjects with PD and controls (*n* = 38)	Arithmetic calculations while listening to loud music (protocol not specified)	*Symptom outcomes: *Eshkol and Wachman coding system for reach-to-grasp movements, duration of forward and backward movements, and emotional state questionnaire (tool not specified)	*Symptom outcomes: *stress was associated with significant deteriorations in mood and reduced hedonistic responsiveness in subjects with PD. Stress did not result in significant differences in each-to-grasp movements between subjects and controls.

Macht et al., 2005 [65]	*Purpose: *to examine patterns of psychological problems in subjects with PD to include symptom frequencies and health outcomes *Sample: *male and female subjects with PD (*n* = 3075)	Not applicable	*Symptoms outcomes: *Inventory of Psychosocial Stress in PD *Health outcomes: *Inventory of Psychosocial Stress in PD and benefits of social support questionnaire (tool not specified)	*Symptom outcomes: *Symptom increases with even small amounts of stress were reported by approximately two-thirds of subjects. Higher stress levels were associated with greater frequencies in depressive moods, sleep disturbances, anxiety, sexual problems, and communication difficulties. *Health outcomes: *higher stress was associated with greater difficulties coping with PD, worsening social relationships, less enjoyment of life, and the need for more psychological support.

PD, Parkinson's disease; FoG, freezing of gait; WAIS-R, Wechsler Adult Intelligence Scale-Revised; SSR, sympathetic skin responses.
